# The role of 25-hydroxyvitamin D deficiency in promoting insulin resistance and inflammation in patients with Chronic Kidney Disease: a randomised controlled trial

**DOI:** 10.1186/1471-2369-10-41

**Published:** 2009-12-10

**Authors:** William G Petchey, Ingrid J Hickman, Emma Duncan, Johannes B Prins, Carmel M Hawley, David W Johnson, Katherine Barraclough, Nicole M Isbel

**Affiliations:** 1 Department of Nephrology, University of Queensland at Princess Alexandra Hospital, Brisbane, Australia; 2 Diamantina Institute for Cancer, Immunology and Metabolic Medicine, University of Queensland, Brisbane, Australia; 3 Centres of Health Research, Princess Alexandra Hospital, Brisbane, Australia

## Abstract

**Background:**

Approximately 50% of patients with stage 3 Chronic Kidney Disease are 25-hydroxyvitamin D insufficient, and this prevalence increases with falling glomerular filtration rate. Vitamin D is now recognised as having pleiotropic roles beyond bone and mineral homeostasis, with the vitamin D receptor and metabolising machinery identified in multiple tissues. Worryingly, recent observational data has highlighted an association between hypovitaminosis D and increased cardiovascular mortality, possibly mediated via vitamin D effects on insulin resistance and inflammation. The main hypothesis of this study is that oral Vitamin D supplementation will ameliorate insulin resistance in patients with Chronic Kidney Disease stage 3 when compared to placebo. Secondary hypotheses will test whether this is associated with decreased inflammation and bone/adipocyte-endocrine dysregulation.

**Methods/Design:**

This study is a single-centre, double-blinded, randomised, placebo-controlled trial. Inclusion criteria include; estimated glomerular filtration rate 30-59 ml/min/1.73 m^2^; aged ≥18 on entry to study; and serum 25-hydroxyvitamin D levels <75 nmol/L. Patients will be randomised 1:1 to receive either oral cholecalciferol 2000IU/day or placebo for 6 months. The primary outcome will be an improvement in insulin sensitivity, measured by hyperinsulinaemic euglycaemic clamp. Secondary outcome measures will include serum parathyroid hormone, cytokines (Interleukin-1β, Interleukin-6, Tumour Necrosis Factor alpha), adiponectin (total and High Molecular Weight), osteocalcin (carboxylated and under-carboxylated), peripheral blood mononuclear cell Nuclear Factor Kappa-B p65 binding activity, brachial artery reactivity, aortic pulse wave velocity and waveform analysis, and indirect calorimetry. All outcome measures will be performed at baseline and end of study.

**Discussion:**

To date, no randomised controlled trial has been performed in pre-dialysis CKD patients to study the correlation between vitamin D status with supplementation, insulin resistance and markers of adverse cardiovascular risk. We remain hopeful that cholecalciferol may be a safe intervention, with health benefits beyond those related to bone-mineral homeostasis.

**Trial registration:**

Australian and New Zealand Clinical Trials Registry ACTRN12609000246280.

## Background

Chronic Kidney Disease (CKD) is an independent predictor of all-cause and cardiovascular (CV) mortality[1]. Whilst it is true that traditional Framingham risk factors are amenable to intervention in CKD, the results of intervention may not be as efficacious as those obtained in the general population[2,3]. Thus, it has been hypothesised that there is a unique milieu established in CKD, which causes excess CV burden by mechanisms that are as yet not fully understood.

Hypovitaminosis D, measured as circulating 25-hydroxyvitamin D (25-OHD), is now widely recognised as a spectrum state, incorporating both deficiency (25-OHD <37 nmol/L) and relative insufficiency (38-75 nmol/L)[4]. CKD is an independent risk factor for hypovitaminosis D3, the prevalence of which negatively correlates with falling estimated glomerular filtration rate (eGFR) from CKD stage 3 onwards[5]. To contextualise this, in a recent audit of our department, the prevalence of hypovitaminosis D3 was found to be 54% in patients with CKD stage 3, with 11% of individuals deficient and 43% insufficient (unpublished data).

Dobnig et al were amongst the first to demonstrate that patients in the general population with lower serum 25-OHD and 1,25-(OH)_2_D (the active metabolite) at baseline are at increased risk of all-cause and cardiovascular mortality[6]. Similar findings have been reported in the incident haemodialysis population, and in CKD stages 3 and 4[7,8]. Furthermore, observational studies demonstrate that in End Stage Kidney Disease (ESKD), patients prescribed active vitamin D compounds appear to have a 26% reduction in mortality, independent of other cardiovascular risks, parathyroid hormone (PTH) and bone mineral markers[9].

There is now emerging mechanistic data from laboratory studies linking vitamin D to pathological processes that could explain cardiovascular risk modulation. These include insulin resistance, inflammation and bone-endocrine dysregulation.

Within the CKD population impaired glycaemic control is highly prevalent[5,10], is associated primarily with an increase in target tissue insulin resistance[11], and is additive to the CV burden [12-14]. Insulin sensitivity in normoglycaemic adults has been shown to be improved with vitamin D supplementation[15], and several small studies involving haemodialysis patients have yielded similar results using 1,25-(OH)_2_D or it's analogues for 4-26 weeks [16-26]. Mechanisms that may explain this improvement include down-regulation of the Renin-Angiotensin-Aldosterone system (RAAS), and possibly up-regulation of the osteocalcin-adiponectin system.

RAAS over-activity is well characterised in vitamin D deficiency[27], although the underlying mechanism for this is not fully understood. Excessive RAAS activity leads to diminished activation of the intracellular insulin receptor substrate-1 (IRS-1) - phosphatidylinositol 3-kinase(PI3-K) signalling pathway. This ultimately decreases translocation of the GLUT-4 glucose transporters to the cell membrane in skeletal muscle; a process integral to augment the action of insulin and enhancing glucose uptake into cells[27,28].

Osteocalcin is a bone protein secreted by osteoblasts, the transcription of which is known to be vitamin D responsive[29]. Additionally, osteoblasts express the vitamin D activating 1α-hydroxylase enzyme (CYP27B1) and *ex vivo *culture with the substrate 25-OHD yields greater mRNA production for osteocalcin than 1,25-(OH)_2_D, highlighting the possible importance of substrate availability[30]. Recent studies have linked osteocalcin to improved glycaemic handling in tissues[31], and osteocalcin knockout mice exhibit marked insulin resistance and an adverse metabolic phenotype[32]. High molecular weight (HMW) adiponectin is an adipokine that is known to improve glycaemic handling, and negatively correlates with insulin resistance[33]. Adiponectin release from adipocytes is stimulated by osteocalcin[32] and thus may be the linking intermediary.

Adverse glycaemic control is also associated with increased inflammatory stress[34,35], as occurs in CKD, and the resultant pro-inflammatory state is associated with atherosclerosis[36,37]. Vitamin D deficiency is associated with an increased inflammatory burden in humans[6], and this can be suppressed using 1,25-(OH)_2_D or its analogues, both *in vitro *and *in vivo *[38-41]. A major factor affecting cytokine production by circulating peripheral blood mononuclear cells (PBMCs) is Nuclear Factor Kappa-B (NfκB), a family of dimeric nuclear transcription factors that when activated are associated with the production of pro-inflammatory cytokines. Various groups have shown that the transcription factor NFκB family is linked to the vitamin D receptor (VDR) signalling pathway [42-44], and that vitamin D inhibits monocyte NFκB p65 activation with down stream suppression of various pro-inflammatory cytokines including Tumour Necrosis Factor alpha (TNFα), Interleukin (IL)-1β, and IL-6[45]. PBMCs also possess the CYP27B1 enzyme[45], which is up-regulated in uraemia but is not responsive to PTH and calcium like the renal isoform[46]. This raises the question whether, like osteoblasts, there may be more avid responses to supplementing the substrate precursor 25-OHD. Unfortunately, to date, Vitamin D intervention trials in the renal literature have predominantly used active 1,25-(OH)_2_D compounds and the results are discrepant, with groups reporting both decreased and no change in PBMC cytokine production, with no obvious correlation with serum cytokine profiles, in either a dose, duration or PBMC specific fashion[41,47-54].

Whilst active vitamin D3 analogues have limited application due to side-effects (hypercalcaemia, hyperphosphataemia), vitamin D3 supplementation (as oral cholecalciferol) is well tolerated at both physiological and supra-physiological doses, is efficacious at raising 25-OHD levels, and is not associated with the same degree of mineral disturbance[55,56].

This study aims to provide prospective data regarding the novel use of cholecalciferol, a safe and effective vitamin D supplement, to ameliorate insulin resistance in patients with CKD. Improvements in insulin sensitivity with cholecalciferol intervention will be assessed for association and possible causation with circulating inflammatory burden and bone/adipocyte-endocrine regulation. This will also be related to vascular function, a surrogate marker for cardiovascular risk.

## Methods/Design

This trial is a double-blinded randomised controlled trial. Informed consent will be obtained from all participants. The study protocol has received approval from the Princess Alexandra Hospital Human Research Ethics Committee (PAH HREC), approval number 2009/097. The project is funded by a Princess Alexandra Hospital Private Practice Trust Fund Research Support Grant and a Neorecormon Research Grant from Roche Products Pty Limited.

### Participants

All patients attending the Nephrology outpatient department at Princess Alexandra Hospital, Brisbane meeting inclusion criteria will be invited to participate. Inclusion criteria include: CKD stage 3 (eGFR[57] 30-59 ml/min/1.73 m^2^); age ≥18 or over; and serum 25-hydroxyvitamin D level <75 nmol/L. Exclusion criteria include: unable or unwilling to give informed consent; pre-menopausal status in females; death or dialysis expected within 6 months; recipient of a transplant; active (<1 month) inflammatory or infectious process or use of anti-inflammatory medication; corticosteroid use (<1 month); oral hypoglycaemic medication use; current or recent (<3 months) use of drugs affecting bone turnover, hepatic metabolism of vitamin D, steroids, or vitamin D containing compounds; malabsorptive state or lactose intolerant; active endocrine disorder (excluding diabetes); serum corrected calcium >2.54 mmol/L or phosphate >1.49 mmol/L.

### Intervention

Randomised patients will receive either 2000IU oral cholecalciferol/day (two 25 μcg tablets, Ostevit-D, Key Pharmaceuticals Pty Limited, NSW) or two placebo tablets/day for a total duration of 6 months. Patients with serum calcidiol levels ≤37 nmol/L cannot be ethically randomised to placebo and will therefore be placed on cholecalciferol at a dose of 2000IU/day with subsequent follow up for 6 months. The non-randomised patients will be analysed primarily by dependent paired sampling, acting as their own control. If, in subsequent analysis, this group is found to have comparable baseline characteristics to the randomised groups, independent analysis will be performed.

### Outcomes

All outcome measures will be performed at baseline and end of study. The primary outcome measure will be a change in the glucose disposal rate as a marker of insulin sensitivity as measured by the hyperinsulinaemic euglycaemic clamp[58]. Secondary outcome measures will include serum PTH, cytokines (IL-1β, IL-6, TNFα), adiponectin (total and High Molecular Weight), osteocalcin (carboxylated and under-carboxylated), and peripheral blood mononuclear cell NFκB p65 binding activity, all of which will be tested on -80°C stored serum (nuclear extract for NFκB) in batch by enzyme-linked immunosorbent assay (ELISA). Brachial artery reactivity, aortic pulse wave velocity and waveform analysis, and indirect calorimetry will also be assessed.

### Sample size and randomisation

Prospective power calculations based on independent t testing indicate that the study will have adequate statistical power (80% probability) to detect a clinically significant increase in insulin sensitivity of 12%, assuming α = 0.05 and a population standard deviation of 10%, if 24 patients are recruited in the study (12 in each group). Using paired t testing for dependent sampling (non-randomised group, baseline to end of study), 8 patients will be needed. From previous experience within the renal unit of CKD trials, it is known that there is a high drop out rate because of progression to dialysis, death, intercurrent co-morbidity and loss to follow up. Allowing for a 20% drop out and using the adjustment factor 1/(1-*v*)^2^, a total of 51 patients will need to be recruited.

Randomisation will be by permuted block and stratified for baseline Body Mass Index (BMI, ≤25 Kg/m^2 ^and >25 Kg/m^2^) and diabetic status. Randomisation will be performed off-site using a validated Microsoft Access Computer based system available through the Centre for Clinical Research Excellence Vascular and Metabolic Health at the University of Queensland. This will generate a unique treatment allocation number which will be ascribed to the patient blindly by the physician. Pharmacy will be un-blinded and dispense trial medication based upon the numeric reference prescription, using unmarked bottles, with generic administration details.

### Statistical methods

Data will be assessed for normality of distribution and transformed as appropriate. The active intervention (Cholecalciferol) and placebo groups will be compared at baseline to determine the effectiveness of randomisation. Euglycaemic clamp data from a healthy, non-uraemic cohort will be supplied by IH and used for comparison. Any differences between the groups that may cause potential confounding (age, sex, ethnicity, BMI, lean:fat mass, eGFR, calcium, phosphate, PTH, inflammatory markers, proteinuria) will be assessed and adjusted for in the analysis by the use of appropriate multivariate techniques.

Statistical analyses will be performed using standard statistical software (Stata Version 10.0, Texas, USA). For parametric continuous data, results will be expressed as mean ± standard deviation, for non-parametric continuous data, results will be expressed as median (interquartile range, IQR), and for categorical data, results will be expressed as frequencies (percentages %). The degree of association between 25-hydroxyvitamin D and the variables of interest will be assessed using Pearson's correlation for continuous normally distributed variables and Spearman's correlation for categorical or non-parametric data. Linear or binary logistic regression will be performed as appropriate and standard regression diagnostics will be performed. Comparison of means for independent samples will be made using: unpaired t-test (parametric data); Mann-Whitney test (non-parametric data); and, Fisher's exact test or Pearson's Chi-square test (categorical data). Comparisons of means for dependent samples will be by paired samples t-test (parametric data); Wilcoxon signed rank test (non-parametric data); and, McNemar's test (categorical data).

No interim analysis is planned, but patients will be monitored for safety by biochemical testing (serum calcium and phosphate) at 3 months. Analysis of insulin resistance related to intervention will be by completed cases only. Secondary hypotheses will be assessed by analysis of available data and intention to treat.

The hypothesis will be accepted if the null hypothesis can be rejected at the 0.05 probability level.

## Discussion

To date, no randomised controlled trial has been performed in pre-dialysis CKD patients to study the correlation between vitamin D status with supplementation, insulin resistance and markers of adverse cardiovascular risk. Given the limitations of active vitamin D compounds, we remain hopeful that cholecalciferol may be a safer intervention, with health benefits beyond those related to bone-mineral homeostasis. Ultimately, this trial will add much needed, controlled, prospective data to the field, and if positive, lead the way for a larger study focussed on addressing patient level outcomes in this challenging population.

## Competing interests

This study has received funding from a Neorecormon Research Grant (Roche Products) and a Private Practice Trust Fund Research Support Grant (Princess Alexandra Research Foundation).

## Authors' contributions

WP conceived the study, wrote the protocol and ethics submission, secured the Roche NRG award and is co-principal investigator on the trial. IH assisted with protocol design and assisted with performing hyperinsulinaemic euglycaemic clamps on participants. JP and EH assisted in the trial design, specifically the components pertaining to end-organ insulin resistance, vitamin D physiology and bone-energy metabolism respectively. CH and DJ advised on the statistical analysis, study design and helped with recruitment of patients. KB recruited patients and gave technical assistance in preparation of the manuscript. NI supervised and assisted with protocol and trial design, secured PAH Foundation Trust funding, and is co-principal investigator for the trial. All authors read and approved the final manuscript.

## Pre-publication history

The pre-publication history for this paper can be accessed here:

http://www.biomedcentral.com/1471-2369/10/41/prepub
